# Physical activity behavior during Covid 19 pandemic among Iranian dwellers in Southern Iran based on planned behavior theory: a SEM analysis

**DOI:** 10.1186/s12889-022-13797-3

**Published:** 2022-07-22

**Authors:** Ali Khani Jeihooni, Fatemeh Jafari, Ramin Shiraly, Tayebeh Rakhshani, Abdolrahim Asadollahi, Hamed Karami

**Affiliations:** 1 Nutrition Research Center, Department of Public Health, School of Health, Shiraz University of Medical Sciences, Shiraz, P. Code:7153675541 Iran; 2 Student Research Committee, Shiraz University of Medical Sciences, Shiraz, Iran; 3 Department of Community Medicine, School of Medicine, Health Behavior Science Research Center, Shiraz University of Medical Sciences, Shiraz, Iran; 4 Department of Health Promotion, School of Health, Shiraz University of Medical Sciences, Shiraz, Iran

**Keywords:** COVID-19, Physical activity, Structural equation modeling (SEM), Theory of planned behavior (TPB)

## Abstract

**Background:**

The COVID-19 pandemic restrictions curtailed physical activity. The current study applied an integrated Theory of Planned Behavior to identify the determinants of physical activity behavior and the processes involved in the COVID-19 pandemic.

**Methods:**

A cross-sectional study was conducted in Shiraz city, Southern Iran, among 2500 people who met the inclusion criteria were included in the study. Data were collected using the demographic information questions and questionnaire based on the Theory of Planned Behavior (TPB) constructs. The Questionnaire via WhatsApp, emails, and SMS was shared. Data analysis was performed using SPSS26 and Amos version 24. Mean and standard deviation was used to describe the data. Also, one-way ANOVA and structural equation analysis were used to analyze the data. The significance level in all the tests was considered to be 0.05.

**Results:**

One thousand one hundred sixty-nine samples (46.8%) said they had been exercising less than 3 days a week, and 47.6% of them did not have any exercise or physical activities (*n* = 1191). The mean score of attitudes, SN, PBC, and intention were 9.38 ± 2.07, 9.27 ± 2.03, 9.32 ± 2.05, and 12.29 ± 2.35, respectively. The effect size values demonstrate the independent variables’ high coefficient of influence on explaining the theoretical model. According to the results, the factors play an important role in samples’ intention (η^2^ ≥ 0.2, *p* ≤ 0.05). The effect size of intention on doing physical activities and exercise during the SARS-CoV-2 pandemic is Eta square = 0.777, which means the measure was high. The obtained model was good based on the main goodness of fit indices (Chi2 = 108.6, df = 25, *n* = 2500, Chi2/df = 4.344, RMSEA = 0.036, AGFI = 0.92, CFI = 0.95, GFI = 0.90, Fornell-Larcker criterion = 0.87, HTMT = 0.89).

**Conclusion:**

The TPB provides a useful framework to explore psychosocial determinants of physical activity behavior during the pandemic and identify key strategies for program planning aimed at improving exercise among people who were already influenced by quarantine and lockdown restrictions.

## Background

At the end of 2019, a new coronavirus (now known as SARS-CoV-2) was discovered in Wuhan, China, and spread worldwide. On January 31, 2020, the World Health Organization labeled the outbreak a worldwide public health emergency of international concern and declared it a global public health emergency [1]. Human-to-human virus transmission occurs through intimate contact with an infected individual and exposure to cough, sneeze, respiratory droplets, or airborne particles that can be inhaled through the nose or mouth [2]. Governments have progressively developed deterrent techniques, such as social separation and other societal restraints that may harm mental and physical health in response to the disease’s epidemic and worldwide threat status [3]. In people at risk for cardiovascular disease, decreased physical activity levels during Covid 19 social isolation may rapidly lead to cardiovascular health issues and untimely death. Even brief inactivity (1 to 4 weeks) is associated with detrimental effects on cardiovascular structure and function and an elevated risk of cardiovascular disease [4]. Physical activity may have a significant role in the care of mild to moderate mental health illnesses, including depression and anxiety, and the symptoms of anxiety and panic disorder generally improve with regular exercise [5]. This topic specifies the function of models and theories of studying behavior in health education, given that human behavior is a reflection of various circumstances and health education is viewed as the focal point of health activities and programs [6]. The idea of planned behavior is one of the theories of behavior modification that can be used to predict and comprehend behavior to improve it [7]. The theory of planned behavior (TPB) has been extensively utilized to analyze and guide health-related activities, such as abstinence from alcohol/smoking and exercise and is impacted and predicted by views (favorable and unfavorable) [8]. This model has the ability to be used in studies related to screening for various diseases [9]. To date, no study has specifically used TPB in relation to Covid19 in Iran. According to this theory, the desire of individuals to engage in a particular activity is impacted by three primary factors: attitude, subjective norm, and perception of behavior control [10]. This idea proposes that a person’s intention to engage in conduct is the closest key driver of behavior. The intention is a person’s motive and desire to attempt to do a behavior [11]. Attitude is best defined as assessing a certain behavior and its perceived effects. Finally, perceived behavioral control (PBC) addresses control beliefs that include barriers and facilitators of behavior performance [12]. In conclusion, TPB demonstrates that intentions are the primary source of behavioral action, although intentions are afterwards recognized by emotional attitudes (anticipated pleasure) and instrumental attitudes (expected usefulness), subjective norm (perceived social pressure), and behavioral control. Excessive conduct is identified [13]. Physical activity, on the other hand, has been demonstrated to be beneficial for mental health, but the significance of physical activity in relation to mental health during social distance owing to the epidemic of covid 19 in the Iranian community is not obvious at this time. Considering the type of study that evaluates the factors affecting the effectiveness of Covid-19 prevention measures during the epidemic in the Philippines [14] and suggests that it introduces the main idea of the TPB model to evaluate the effectiveness of COVID-19 measures in other countries. This structure with a larger sample size than the same study was selected and implemented for physical activity. As a result, the goal of this study was to explore the behavior of physical activity during the Covid 19 pandemic in Shiraz using the idea of planned behavior.

## Methods

This is a cross-sectional study with a total research population of persons over 18 living in Shiraz city, Southern Iran. According to Chirico’ study [15], the sample size is about 2500 Iranian citizens using convenience sampling approach. Information was submitted for sampling through social media platforms like as Instagram and Facebook, as well as through Shiraz University of Medical Sciences’ official channels, and 2500 participants were considered in this study from June 2020 to September 2021. A social media platform i.e. WhatsApp was utilized to make the survey available for anyone to answer. Specifically, the survey was made available for anyone who has an account and is active on this social media. The online survey is fully anonymous and cannot be traced back to the respondents’ identities, and their anonymity is guaranteed. Hiking routes and cycling tracks were created in collaboration with the municipality to encourage their cooperation. Individuals expressed their willingness to participate before answering the questions. A brief description of the study and its objective was also supplied. Participants were informed that the online survey is fully anonymous and cannot be tracked in terms of the respondents’ identities, and their confidentiality is guaranteed.

Inclusion criteria: reading and writing literacy, possession of a cell phone, People over 18 (male and female), Shiraz residents. Fill out the informed consent form.

Criteria for exclusion: Reluctance to take part in the study.

Attitudes, subjective norms, PBC, and intention from the TPB were measured using measures utilized in previous studies [16].

The initial set of demographic information questions comprised age, gender, occupation, education, marital status, insurance coverage, recent travel, and outdoor living space measurements.

The second component included the Behavioral Regulation Questionnaire in Sport (BREQ-3) and the Autonomy Index Questionnaire (RAI), which yielded scores based on the questionnaire’s subscales. Positive scores suggest greater relative independence. Each item in the attitudes construct was preceded by “I believe conducting a physical exercise during this quarantine period is:” consisting of three items with responses supplied on 7-point semantic differential scales with the bipolar adjectives “wrong right,” “disadvantageous-advantageous,” and “useless-useful.”

Subjective Norms were determined by asking participants, for example, “would you prefer me to conduct physical exercise during this quarantine period?” Responses were provided on a 7-point Likert type scale (1 = “strongly disagree” and 7 = “strongly agree”). The item scores were combined into a single score, with higher values indicating greater normative societal pressure toward the activity.

PBC was assessed with three items (e.g., “I’m certain I can exercise during this quarantine period”) and responses on a 7-point Likert-type scale (e.g., 1 = “no control” and 7 = “strong control”). Item scores were combined into a single score, with higher values indicating better-perceived confidence in the behavior.

Finally, respondents were asked to express their intention on a 7-point Likert type scale (1 = “strongly disagree” and 7 = “strongly agree”) for four statements (e.g., “I expect to conduct physical exercise during this quarantine period”). Item scores were combined into a single score, with higher values indicating greater intent to engage in the behavior.

Cronbach’s alpha internal consistency approach was used to determine the reliability of the questions [15]. The final section consisted of seven-choice Likert-scale questions on the components of the theory of planned behavior, including three questions on attitudes, three on abstract, subjective norms, three on perceived behavioral control, and four on physical activity intention.

A total of 12 experts (outside the research team) were consulted for content validity in the areas of health education and health promotion (10 individuals) and infectious disease specialists (2 individuals).

Using structural equation modeling, relationships among the constructs were examined (SEM). Multivariate structural equation models (SEMs) combine different analytical methods (factor analysis and multiple regression analyses) to study and assess the relationships between latent and measured variables (measurement model) and between latent variables (structural model) while simultaneously accounting for measurement errors [17].

.Version 26 of SPSS and version 24 of Amos were used to undertake data analysis (IBM Co., Ann Arbore). The data were described by the mean and standard deviation. In addition, one-way ANOVA and structural equation analysis were applied to the data analysis. The level of significance for each test was deemed to be 0.05.

## Results

Participant characteristics of the sample are presented in Table 1. Of the 2500 participants, 41.8 and 58.2% were male and female, respectively. The mean age of participants was 41.79 ± 15.21, 85.7% were married and 24% in other yards, 58.5% received less than 104 US$ per month, and 1226 (49%) of participants had a high school diploma. (58.4%) of them lived in a house between 71 m^2^ and 100 m^2^.* The next question was the availability of outdoors such as balcony, terrace, backyard, and garden at home. About 71.4% of samples said their house has a terrace and use it (*n* = 1785). A question was about the time of physical activity during the coronavirus pandemic, and 1169 samples (46.8%) said they had been exercising less than 3 days a week, and 47.6% did not exercise or do physical activities (*n* = 1191). The mean score of attitude, SN, PBC, and intention were 9.38 ± 2.07, 9.27 ± 2.03, 9.32 ± 2.05, and 12.29 ± 2.35, respectively. According to Table 1, there is no relationship between demographic variables and physical activity, except for the outdoor access variable.

**Table 1:** Demographic characteristics of samples

Variables	Physical activity				
	Nothing	≤ 3 days	4–5 days	≤ 6 days	***p*** value
**Gender**					
Male	513 (49.1%)	479 (45.9%)	29 (2.8%)	23 (2.2%)	0.474
Female	678 (46.6%)	690 (47.4%)	52 (3.6%)	36 (2.5%)	
**Literacy**					
Elementary	26 (44.8%)	29 (50%)	2 (3.4%)	1 (1.7%)	
2nd School	372 (47.1%)	369 (46.8%)	27 (3.4%)	21 (2.7%)	
High School	594 (48.5%)	565 (46.1%)	40 (3.3%)	27 (2.2%)	
Graduated	199 (46.6%)	206 (48.2%)	12 (2.8%)	10 (2.3%)	0.995
**Occupation**					
Employee	194 (43.1%)	231 (51.3%)	16 (3.6%)	9 (2%)	
worker	97 (48%)	93 (46%)	10 (5%)	2 (1%)	
free	370 (48.9%)	341 (45.1%)	26 (3.4%)	19 (2.5%)	
Housewife	297 (47.7%)	293 (47%)	17 (2.7%)	16 (2.6%)	
other	233 (49.7%)	211 (45%)	12 (2.6%)	13 (2.8%)	0.526
**Marital Status**					
Non-maried	197 (45.5%)	212 (49%)	10 (2.3%)	14 (3.2%)	0.247
Married	994 (48.1%)	957 (46.3%)	71 (3.4%)	45 (2.2%)	
**Income** (per month)					
≤ 104 US$	500 (48.2%)	483 (46.5%)	29 (2.8%)	26 (2.5%)	0.712
≥ 105 US$	691 (47.3%)	686 (46.9%)	52 (3.6%)	33 (2.3%)	
**Area of the Home** (m^2^)					
≤ 70	411 (50.3%)	354 (43.3%)	27 (3.3%)	25 (3.1%)	
71–100	421 (45.6%)	460 (49.8%)	27 (2.9%)	16 (1.7%)	
≤ 101	359 (47.4%)	354 (46.7%)	27 (3.6%)	18 (2.4%)	0.286
**Availability of Outdoors**					
Balcony	159 (50.5%)	143 (45.4%)	8 (2.5%)	5 (1.6%)	
Terrace	839 (47%)	843 (47.2%)	68 (3.8%)	35 (2%)	
Garden	137 (48.1%)	128 (44.9%)	4 (1.4%)	16 (5.6%)	
Nothing	43 (45.3%)	50 (52.6%)	0 (0%)	2 (2.1%)	0.004

According to Table 2, the fixed effect ANOVA results were obtained for TPB constructs. The effect size values demonstrate the independent variables’ high coefficient of influence on explaining the theoretical model.

**Table 2:** Fixed Effect ANOVA Results for TPB Constructs

Ffixed Factors	***df***	***F***	Effect Size^a^	Partial η^**2**^ 90% CI [LL,UL]	Sig.
	1. Intention				
Attitude	2488	9.551	.241	[.184, .351]	.000^*^
SN	2490	5.607	.220	[.137, .281]	.000^*^
PBC	2490	5.127	.218	[.134, .288]	.000^*^
	2. Physical Activity				
Intention	2487	721.39	.777	[.654, .891]	.000^**^

^a^*Using Eta square (η*^*2*^*),* ^*^ *p < .05,* ^**^ *p < .01*

**Fig. 1:**
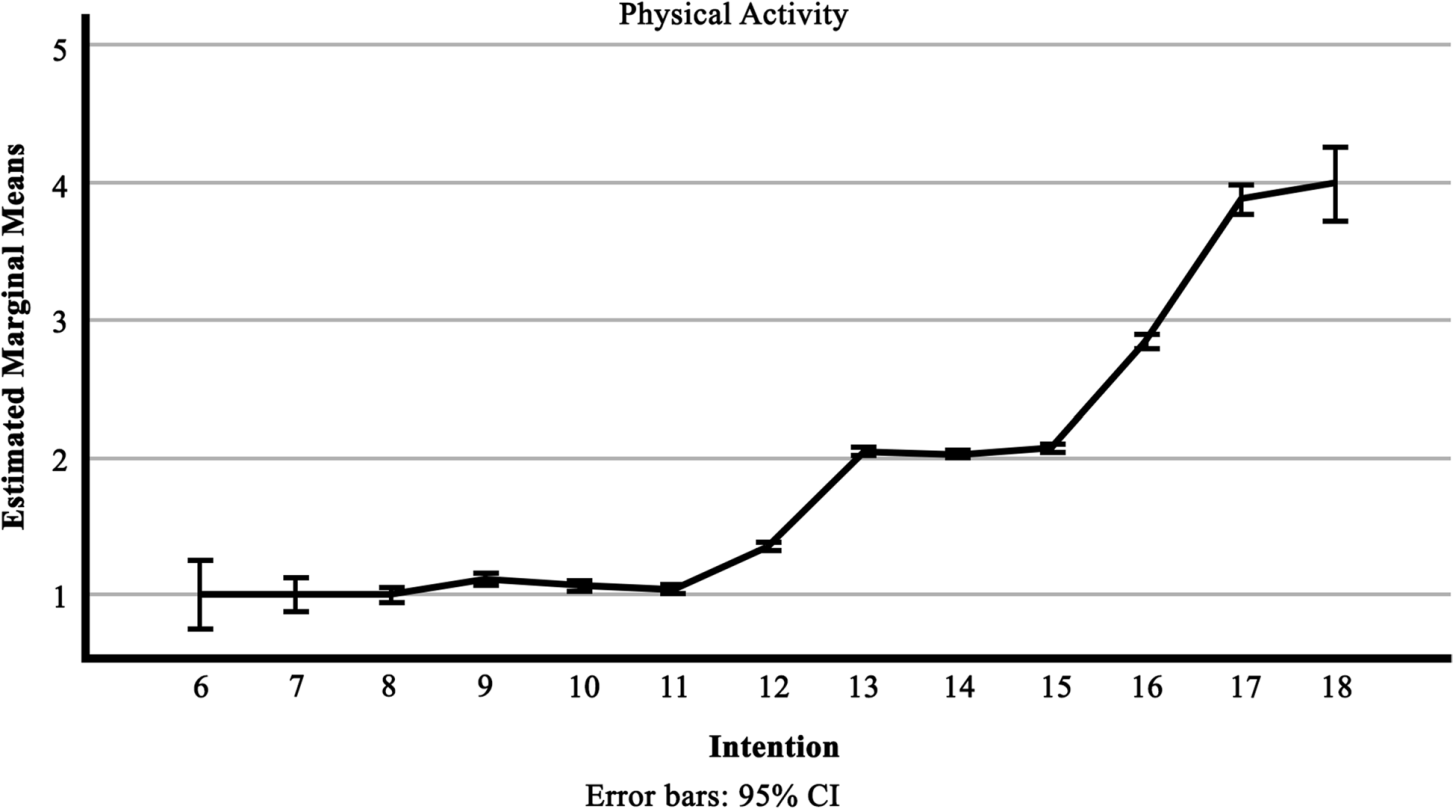
Effectiveness of intention on physical activiy of samples (*n* = 2500)

**Fig. 2:**
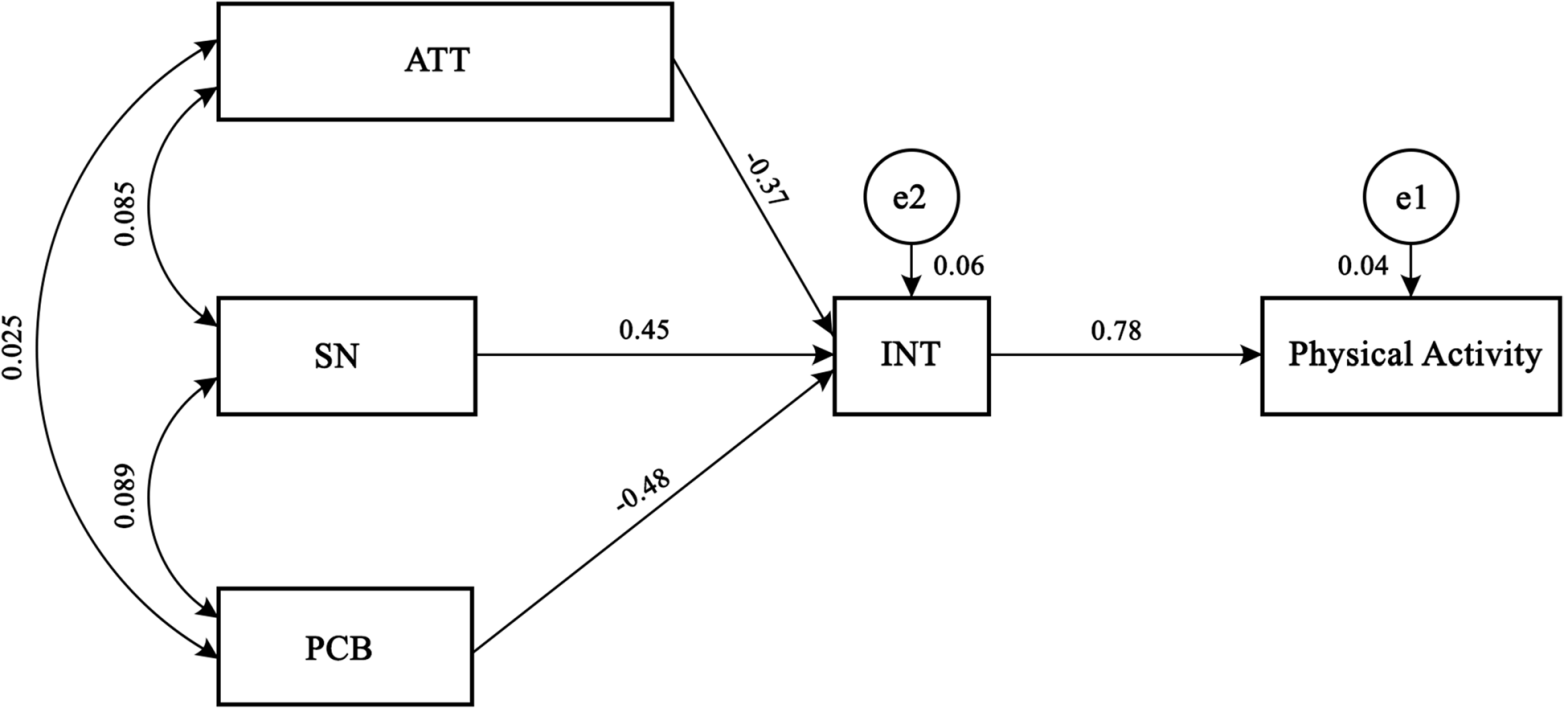
Final Structural Equation Model of the Study (*n* = 2500). Abbreviations: ATT = Attitude, SN = Subjective Norms, PBC = Perceived Behavior Control, INT = Intention

According to the results, the factors play an important role in samples' intention (η^2^ ≥ 0.2, *p* ≤ 0.05). The effect size of intention on doing physical activities and exercise during the SARS-CoV-2 pandemic is Eta square = 0.777, and it means the measure was high (See Fig. 1, Spearman's rho coefficient = .84, *p* < .01)The three constructs of the theoretical model and demographic variables were entered into the model using the SEM. Out of 3 extraction models, according to Table 3 and Fig. 2, the following model with the highest fit index was obtained.

**Table 3:** Result of three construct of theoretical model along with demographic variables by SEM

Examined Paths	Mean	SD	Standardized Path Coefitients
**ATT**	9.38	3.07	
ATT ➔ INT			−0.37
**SN**	9.27	2.03	
SN ➔ INT			0.45
**PBC**	9.32	2.05	
PBC ➔ INT			−0.48
**INT**	12.29	2.35	
INT ➔ Phy.Act.			0.78
**Phy.Act.** ^a - b^			
Level 1	1191 (47.6)		
Level 2	1169 (46.8)		–
Level 3	140 (5.6)		
**Model Fit**	Chi2 = 108.6, df = 25, *n* = 2500, Chi2/df = 4.344, RMSEA = 0.036, AGFI = 0.92, CFI = 0.95, GFI = 0.90, MSV ≥ 0.78, Fornell-Larcker criterion = 0.87, HTMT = 0.89.		

Note: *ATT* Attitude, *SN* Subjective norms, *PBC* Perceived behavioral control, and INT = intention

* *p* ≤ 0.05

^a^Level 1 = No physical activity, level 2 = Less than 3 days per week, and level 3 = More than 4 days per week

^b^Frequencies (%)

According to the Fig. 2, the obtained model was good based on the main goodness of fit indices (Chi2 = 108.6, df = 25, *n* = 2500, Chi2/df = 4.344, RMSEA = 0.036, AGFI = 0.92, CFI = 0.95, GFI = 0.90).

Discriminant validity is referring to being different between constructs from one another empirically. It can be evaluated by using cross-loading of indicator i.e. the Fornell-Larcker criterion and Heterotrait-monotrait (HTMT) ratio of correlation and the values were 0.87 and 0.89 respectively. Also, the composite reliability (CR) index i.e. maximum shared squared variance (MSV) was calcualetd ans it was a good reliability for all factors (all above 0.78).

Furr (2011) indicated that the fit indices should have standardized loadings of 0.80 and more [18]. The model also has good fit indices (see Fig. 2).

## Discussion

The current study sought to examine the relations between social-cognitive variables and PA behavior during the COVID-19 pandemic, using SEM to test a two-component model of the TPB for PA in a sample of Iranian adults. As expected, the TPB constructs were significantly correlated to engaging in physical activity during the pandemic. This finding suggests that TPB applies to PA behaviors in the pandemic situation. Participants with higher intentions were more likely to report higher physical activity levels. There were significant model pathways to the intention from ATT, SN, and PBC. However, the PBC seems to have the strongest indirect effect on PA levels. These findings align with previously published TBP studies of PA behavior during the pandemic [19, 20]. In another study that aimed to examine the factors affecting the perceived effectiveness of prevention using TPB theory, they concluded that according to PBC, people with sufficient knowledge stay at home more and follow the implementation of quarantine in the community [14]. A meta-analysis by McEachan et al. demonstrated that, regardless of the kind of health behavior, ATT and PBC are the strongest variables associated with behavioral intention [21]. Also, Lau et al. stated that factors derived from TPB are significantly associated with behavioral intention [22]. In a similar study by Prasetyo et al. it was indecated that intention to follow was significantly affected by PBC, SN, and ATT [14]. Moreover, based on the results of this study, direct effects showed that the perception of COVID-19 did not have a significant direct effect on attitude, but its indirect effect was significant, and our findings support that PBC and ATT have a more powerful influence on physical activity engagement in the pandemic time than SN.

Based on our results, approximately half (48%) of Iranian adults reported no physical activity or exercise during the pandemic, and just over 5% of participants reported exercising more than 3 times a week. World Health Organization (WHO) recommends that people aged 18 to 64 engage in at least 150 minutes of moderate PA per week [23]. Pre-pandemic data suggest that most adults do not follow recommended guidelines. For example, the Center for Disease Control (CDC) estimates that more than 60% of U.S. adults did not engage in the recommended amount of PA before the pandemic, and almost 25% of people were not active at all [24]. COVID-19 pandemic has negatively affected all PA levels among adult population [25]. Our findings raise serious concerns about the negative effects of the pandemic on PA behaviors of Iranian adults as it demonstrated that more than 90% of Iranian adults do not meet the recommended guideline. This issue suggests a large-scale behavioral change.

Overall, our findings provide better insight into understanding theory-grounded psychosocial variables associated with physical activity behavior during the pandemic situation. Across all the TPB constructs, the most notable themes that emerged included PBC and SN. Therefore, pandemic response policies should address these issues, which may be pivotal for program planning.

There are some limitations to this study that should be noted. This was a cross-sectional survey by which changes in the theoretical mediators and PA behavior over time can not be evaluated as this evaluation needs a longitudinal study design. Online data collection restricts the generalizability of the results, as sampling bias is unavoidable in web-based surveys. Anxiety about COVID-19 has been suggested as a significant factor that regulates physical activity intention and participation during the pandemic [21]; however, the current study did not assess this variable among the studied population.

## Conclusion

In conclusion, the TPB provides a useful framework to explore psychosocial determinants of physical activity behavior during the pandemic and identify key strategies for program planning aimed at improving exercise among people who were already influenced by quarantine and lockdown restrictions.

## Data Availability

The datasets used and/or analyzed during the current study are available from the corresponding author upon reasonable request.

## Abbreviations

ATT: Attitude
SN: Subjective Norms
PBC: Perceived Behavior Control
INT: Intention
SEM: Structural Equation Model
PA: Physical Activity
TPB: Theory of Planned Behavior
